# Differential Expression Profiling of Long Noncoding RNA and mRNA during Osteoblast Differentiation in Mouse

**DOI:** 10.1155/2018/7691794

**Published:** 2018-03-22

**Authors:** Minjung Kim, Youngseok Yu, Ji-Hoi Moon, InSong Koh, Jae-Hyung Lee

**Affiliations:** ^1^Department of Life and Nanopharmaceutical Sciences, Kyung Hee University, Seoul, Republic of Korea; ^2^Department of Maxillofacial Biomedical Engineering, School of Dentistry, and Institute of Oral Biology, Kyung Hee University, Seoul, Republic of Korea; ^3^Department of Physiology, College of Medicine, Hanyang University, Seoul, Republic of Korea; ^4^Department of Biomedical Informatics, Hanyang University, Seoul, Republic of Korea

## Abstract

Long noncoding RNAs (lncRNAs) are emerging as an important controller affecting metabolic tissue development, signaling, and function. However, little is known about the function and profile of lncRNAs in osteoblastic differentiation in mice. Here, we analyzed the RNA-sequencing (RNA-Seq) datasets obtained for 18 days in two-day intervals from neonatal mouse calvarial pre-osteoblast-like cells. Over the course of osteoblast differentiation, 4058 mRNAs and 3948 lncRNAs were differentially expressed, and they were grouped into 12 clusters according to the expression pattern by fuzzy c-means clustering. Using weighted gene coexpression network analysis, we identified 9 modules related to the early differentiation stage (days 2–8) and 7 modules related to the late differentiation stage (days 10–18). Gene ontology and KEGG pathway enrichment analysis revealed that the mRNA and lncRNA upregulated in the late differentiation stage are highly associated with osteogenesis. We also identified 72 mRNA and 89 lncRNAs as potential markers including several novel markers for osteoblast differentiation and activation. Our findings provide a valuable resource for mouse lncRNA study and improves our understanding of the biology of osteoblastic differentiation in mice.

## 1. Introduction

Ossification is a tightly regulated process which is performed by specialized cells called osteoblasts differentiated from mesenchymal progenitors. The osteoblast differentiation process is regulated by several key factors and signaling pathways. Runt-related transcription factor 2 (Runx2) is the master switch in the commitment of mesenchymal progenitors to osteoblast lineage [1]. Runx2 is affected by several upstream regulators such as the Wnt/Notch system, Sox9, Msx2, and hedgehog signaling as well as by cofactors such as Osx and Atf4 [1–4]. A few paracrine and endocrine factors, including bone morphogenetic proteins (BMP) and parathyroid hormone, serve as coactivators. Vitamin D and histone deacetylase enzymes coordinate this process more finely [1].

Long noncoding RNAs (lncRNAs) are a class of RNA transcripts longer than 200 nucleotides, lacking open reading frames and protein-coding possibilities [5]. Tens of thousands of lncRNAs have been identified in mammalian genomes in recent decades [6]. Recently, in many studies, lncRNA has emerged as an important regulator in a variety of biological processes, such as epigenetic regulation, chromatin remodeling, genomic imprinting, transcriptional control, and pre-/posttranslational mRNA processing [7–11]. In terms of osteogenesis, several lncRNAs have been found to act as key regulators. One such example is maternal expression gene 3 (MEG3) regulating the expression of Bmp4, Runx2, and Osx in human mesenchymal stem cells [12, 13]. Antidifferentiation ncRNA (ANCR) inhibits Runx2 expression in association with the enhancer of zeste homolog 2 (EZH2); thus, downregulation of ANCR promotes osteoblast differentiation through modulation of EZH2/Runx2 [14].

In order to fully understand the lncRNA biology including its role in osteogenesis, it is necessary to characterize the expression pattern of lncRNA during osteoblast differentiation. In this study, we analyzed RNA-sequencing datasets obtained at nine different time points in the osteoblast differentiation of preosteoblasts isolated from neonatal mouse calvaria, using various bioinformatic approaches. We focused on identifying differentially expressed lncRNAs and mRNAs throughout the process and finding potential markers that exhibited significant changes in expression during the osteoblast differentiation.

## 2. Materials and Methods

### 2.1. RNA-Seq Data Processing and Analysis of Differential Gene Expression

We analyzed the RNA-Seq data generated by Kemp et al. [15] (GSE54461, nine time points: 2, 4, 6, 8, 10, 12, 14, 16, and 18 days of osteoblast differentiation). All sequencing reads were aligned to the mouse genome reference (mm10) using the GSNAP alignment tool [16]. Ensembl release 74 annotations were used to measure gene expression. In the case of lncRNAs, NONCODE v4 (http://www.noncode.org) annotations were used. Since NONCODE v4 annotations were based on mm9 mouse genome assembly, the positions of lncRNAs of NONCODE were converted from mm9 to mm10 using the LiftOver utility in UCSC. To assess gene expression, RPKM (reads per kilobase of exon per million mapped reads) values were calculated [17]. Hierarchical clustering of genes expressed in samples at the nine time points was performed using the flashClust R package. To establish the differences in gene expression patterns among nine time points of osteoblast differentiation, we performed differential gene expression analysis using the R package DESeq [18]. The false discovery rate (FDR) was controlled by adjusting *p* values using the Benjamini–Hochberg algorithm. Differentially expressed genes were defined as those with FDR less than 5% with an absolute value of fold change ≥ 2. Similar differential expression analyses were performed for lncRNAs.

### 2.2. Time Series Analysis of Differential Gene Expression

R package DESeq [18] and edgeR [19] were employed to identify genes that were differentially expressed across the differentiation time period to a significant extent, designated as time series genes. We selected time series genes that displayed significant differential expression with FDR < 5% in both DESeq and edgeR, absolute fold change ≥ 2 (between day 2 and at least one other time point), and maximum RPKM ≥ 3 across the time series. Details of the methods for each package are described in the supplementary methods. Similarly, time series expression tests were conducted for lncRNAs. Using the RPKM of each time series gene and lncRNA, principal component analysis (PCA) was performed with the aid of the “prcomp” module in R.

### 2.3. Time Series Gene Clustering

The time series genes identified were clustered using the R package Mfuzz [20] that performs soft clustering based on the fuzzy c-means algorithm. The advantage of soft clustering is that the algorithm clearly reflects the strength of association of an individual gene with a cluster. Average RPKM values (triplicates at each time point) of individual genes were employed as input values for Mfuzz clustering. The number of clusters was set to 12 and the fuzzifier coefficient, M, to 1.5. Heat maps of the clusters were drawn using the R module “heatmap.2” in the “gplots” package [21].

### 2.4. Weighted Gene Coexpression Network Analysis

Gene coexpression network analysis was performed using the R package “WGCNA” [22]. Details of the methods for constructing gene coexpression network analysis are described in the supplementary methods.

### 2.5. Gene Ontology (GO) Term and KEGG Pathway Enrichment Analysis

GO terms of each gene were obtained from Ensembl BioMart and KEGG pathways from the KEGG PATHWAY database. Details of the procedure to conduct the enrichment analysis are described in the supplementary methods.

### 2.6. Analysis of Motif Enrichment

MEME Suite 4.12.20 [23] and the HOmo sapiens COmprehensive MOdel COllection (HOCOMOCO) v11 mouse transcription factor database [24] were used for the identification of motifs in the promoter regions on the lncRNAs identified as potential markers. Details of the procedure to perform the motif enrichment analysis are described in the supplementary methods.

## 3. Results and Discussion

For convenience, the analysis results of mRNA and lncRNAs were described separately. The overall expression pattern was described first, followed by time series analysis, generation of modules by weighted gene coexpression network analysis, and identification of markers for osteoblast differentiation (Supplementary Figure 1).

### 3.1. Expression Profile of mRNA during Osteoblast Differentiation

**Figure 1:**
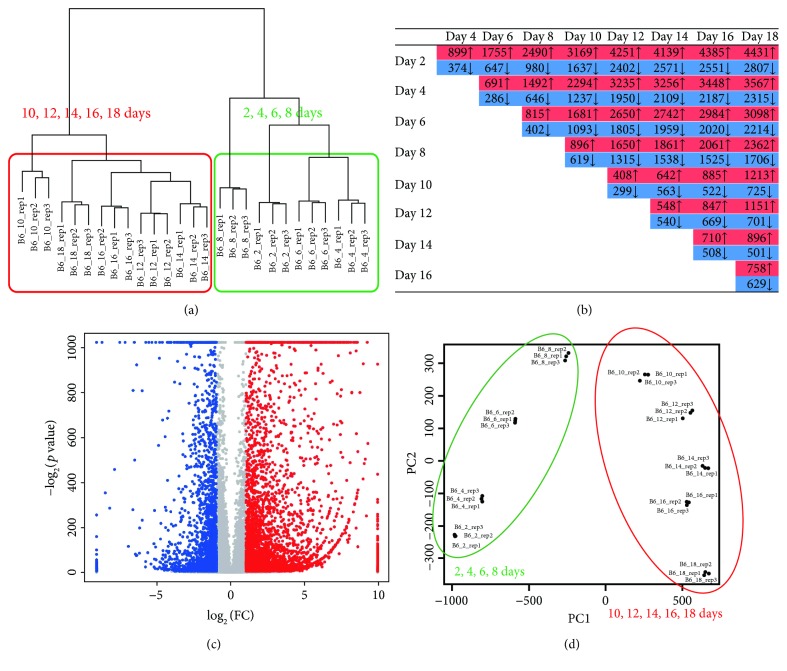
Global expression patterns of genes at the nine time points of osteoblast differentiation. (a) Hierarchical clustering of the transcriptome over the time period of osteoblast differentiation. All known genes with RPKM values ≥ 3 were used for analysis. (b) Number of genes showing up- or downregulation during osteoblast differentiation (fold change ≥ 2 or ≤0.5, FDR < 0.05). (c) Volcano plot showing differentially expressed genes in red and blue. The *x*- and *y*-axes represent the magnitude of fold changes (log_2_ transformed) and adjusted *p* value (−log_2_) by Benjamini-Hochberg correction, respectively. FC = fold change (d18/d2). (d) Principal component analysis (PCA) of 4058 time series gene expression profiles for different samples (three replicates at each time point of osteoblast differentiation).

A total of 12 to 33 million reads (at each time point) were processed and mapped against mouse genome reference (mm10) sequence, and uniquely mapped reads (89.01–90.74% of the total reads) were used for further analysis (Table 1). Based on Ensembl release 74 annotations, a total of 28,582 genes had at least one read for whole RNA-Seq datasets. By hierarchical clustering [25], two distinct clusters were generated for the early (2, 4, 6, and 8 days) and late (10, 12, 14, 16, and 18 days) differentiation stages (Figure 1(a)). As expected, the number of differentially expressed genes increased with time (Figure 1(b)).

Comparing the expression of individual genes at different time points using all possible combinations, 46% of the mapped genes (13,130 out of 28,582) were differentially expressed over time (fold-change difference ≥ 2, FDR < 0.05). For example, a comparison of gene expression between the two time points selected in the early and late stages (day 2 and day 18) is shown in Figure 1(c). A total of 7238 genes were differentially expressed between days 2 and 18 (4431 upregulated and 2807 downregulated on day 18). GO and KEGG pathway analyses revealed that genes upregulated on day 18 were strongly associated with osteogenesis processes, such as “extracellular matrix binding,” “positive regulation of bone mineralization,” “collagen type I,” “elevation of cytosolic calcium ion concentration,” “bone mineralization involved in bone maturation,” and “positive regulation of Wnt receptor signaling pathway” (Supplementary Table 1). On the other hand, genes downregulated on day 18 were associated with cell proliferation processes, such as “cell division,” “G1/S transition of mitotic cell cycle,” “regulation of cell cycle,” “M phase of mitotic cell cycle,” and “DNA replication and response to DNA damage stimulus” (Supplementary Table 2).

### 3.2. Dynamic Changes of mRNA Expression over Time of Osteoblast Differentiation

During the differentiation process, several genes are dynamically expressed via complex regulatory mechanisms. To characterize temporal gene expression changes and patterns, we identified genes that were differentially expressed across the time course of osteoblast differentiation. A number of rules were applied to establish significant differential expression of genes at different time points: (1) statistical significance of temporal gene expression changes assessed via edgeR and DESeq methods (FDR < 0.05), (2) absolute fold change between day 2 and at least one other time point ≥ 2, and (3) maximum RPKM across the time series ≥ 3.

In total, 4058 genes were significantly differentially expressed during osteoblast differentiation. They were associated with osteogenesis processes, such as “extracellular matrix organization,” “osteoblast proliferation,” “osteoblast differentiation,” “positive regulation of osteoblast differentiation,” and “actin cytoskeleton and bone mineralization.” Principal component analysis (PCA) of these genes revealed that 95% of the variations in gene expression could be explained by the first two principal components (PCs) (Supplementary Figure 2) and that the PCs dominantly separate the datasets according to differentiation stage (days 2–8 versus days 10–18) (Figure 1(d)). To evaluate the osteoblast differentiation stages in the current dataset, we have checked the expression level of the twelve mature osteoblast markers (Bglap, Ibsp, Dmp1, Col13a1, Pthr1, Lifr, Bambi, Dlx3, Hnf1a, Phex, Ptgis, and Cdo1) described in Kalajzic et al. [26]. The nine genes, except three genes (Pthr1, Bambi, and Hnf1a), exhibited very similar expression patterns and showed the highest expression at day 18, which indicate that the cells at day 18 were mature osteoblasts (Supplementary Figure 3).

Osteoblasts undergo several stages before maturation and mineralization of bone matrix. Differentiation of osteoblasts, both in vitro and in vivo, is divided into three stages: (1) cell proliferation, (2) matrix maturation, and (3) matrix mineralization [27]. Here, we examined differences in gene expression patterns at the early and late osteoblast differentiation stages. Based on the current study, gene expression profile analysis demonstrated that osteoblast differentiation could be clearly subdivided into two stages. The early stage corresponded to “cell proliferation” based on the Stein and Jane definition [27]. However, we could not separate the late differentiation stage into “matrix maturation” and “matrix mineralization” stages based on the hierarchical clustering and PCA analysis. In addition, the division of osteoblast differentiation into two stages (early and late stages) is observed in the time series gene clustering analysis and weighted gene coexpression network analysis described below.

### 3.3. Clustering Analysis of Time Series Genes

**Figure 2:**
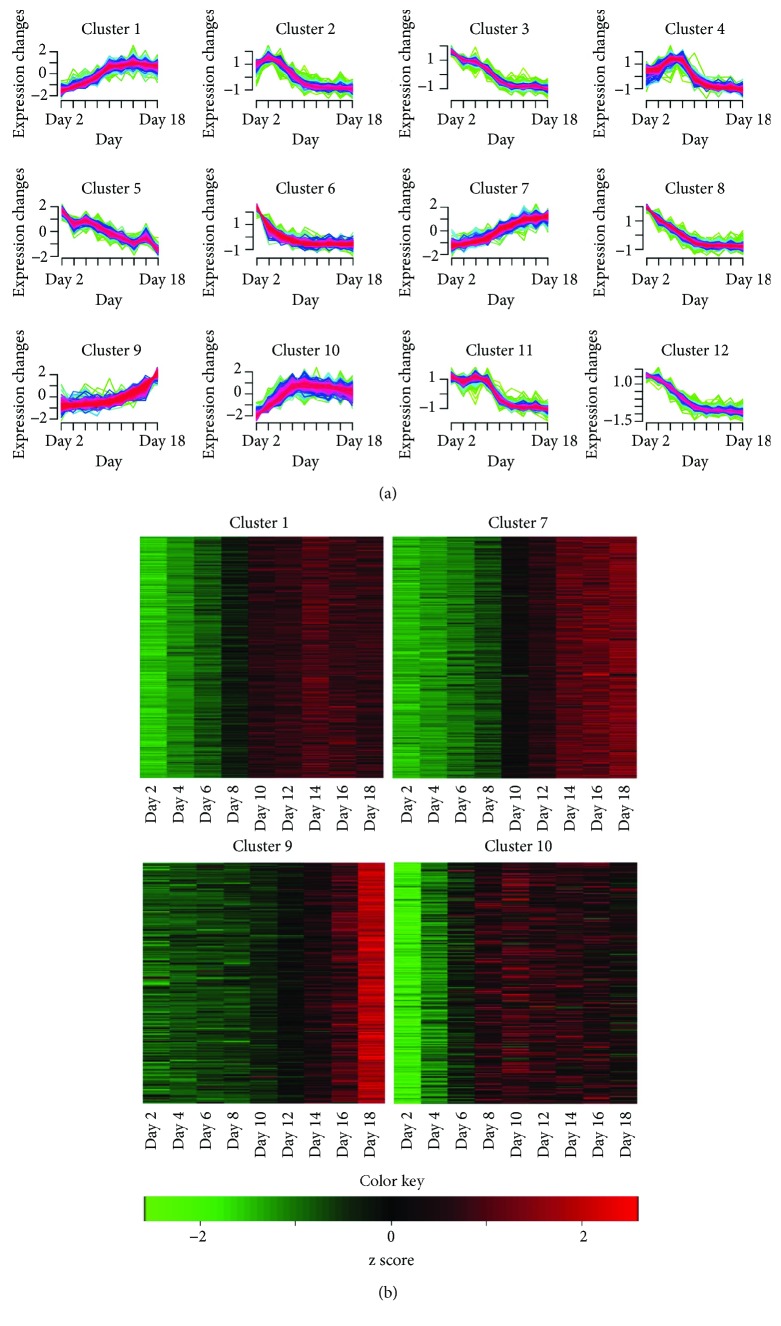
Clustering analysis of time series genes during osteoblast differentiation. (a) Soft clusters of 4058 time series gene expression data using Mfuzz. The numbers on the *x*-axis (time, 1~9) correspond to the nine time points of osteoblast differentiation (days 2, 4, 6, 8, 10, 12, 14, 16, and 18). (b) Heat maps and representative gene expression profiles (RNA-Seq) of four major clusters. The color key indicates gene expression values.

Differentially expressed genes identified by time series analysis were clustered according to their expression profiles (RPKM values) using the fuzzy c-means algorithm implemented in R Mfuzz package. Genes with similar time-specific expression patterns were clustered into 12 groups, each containing 191–570 genes (Figure 2(a)). Eight among the 12 clusters (clusters 2, 3, 4, 5, 6, 8, 11, and 12) showed high expression patterns at the early osteoblast differentiation stage. Functional enrichment analysis for each cluster showed that these 8 clusters were highly enriched for genes related to cell proliferation processes. On the other hand, genes included in the remaining 4 clusters (1, 7, 9, and 10) showed high expression patterns at the late osteoblast differentiation stage. These clusters included several genes associated with functional roles in osteogenesis, such as “extracellular matrix organization,” “osteoblast differentiation,” “metabolic process,” “collagen fibril organization,” “Wnt-protein binding,” and “skeletal system development.”

We focused on clusters 1, 7, 9, and 10, each containing 511, 570, 243, and 297 genes with high expression patterns in the late differentiation stage (Figure 2(b)). Assessment of individual genes within the clusters showed that several are involved in osteogenesis. For example, cluster 1 contained Fgfr2 that plays an essential role in skeletal development [28], cluster 7 contained Bmp4 that promotes formation of the bone and cartilage by stimulating differentiation processes of osteoblasts [29], and cluster 9 contained Fgf18 that has been shown to stimulate the proliferation of cultured mouse primary osteoblasts [30]. Expression levels of Fgfr2, Bmp4, and Fgf18 were particularly high between 12 and 18 days of the osteoblast differentiation process.

We determined the common function among the three clusters through GO and KEGG enrichment analysis. As shown in Table 2, the statistically significant GO terms in the clusters 1, 7, and 9 were “collagen metabolism,” “subset of extracellular matrix,” “regulation of osteoblast differentiation,” and “Wnt signaling” which is known to be involved in the regulation of osteoblast lineage cells [31]. The enriched pathways in the KEGG analysis in the clusters 1, 7, and 9, were “cell adhesion,” “cytokine-cytokine receptor interaction,” and “PPAR signaling pathway” which is functionally associated with bone metabolism [32]. In the case of the cluster 10, “extracellular matrix” is the only statistically significant GO/KEGG term. This indicates that the genes belonging to the three clusters (1, 7, and 9) are highly correlated with the late osteoblast stage but the cluster 10 is less correlated with the late osteoblast stage. Hence, together with the coexpression network analysis described below, we used the genes belonging to the three clusters (1, 7, and 9 except 10) to find biomarkers of osteoblast differentiation.

### 3.4. Construction of a Gene Coexpression Network

**Figure 3:**
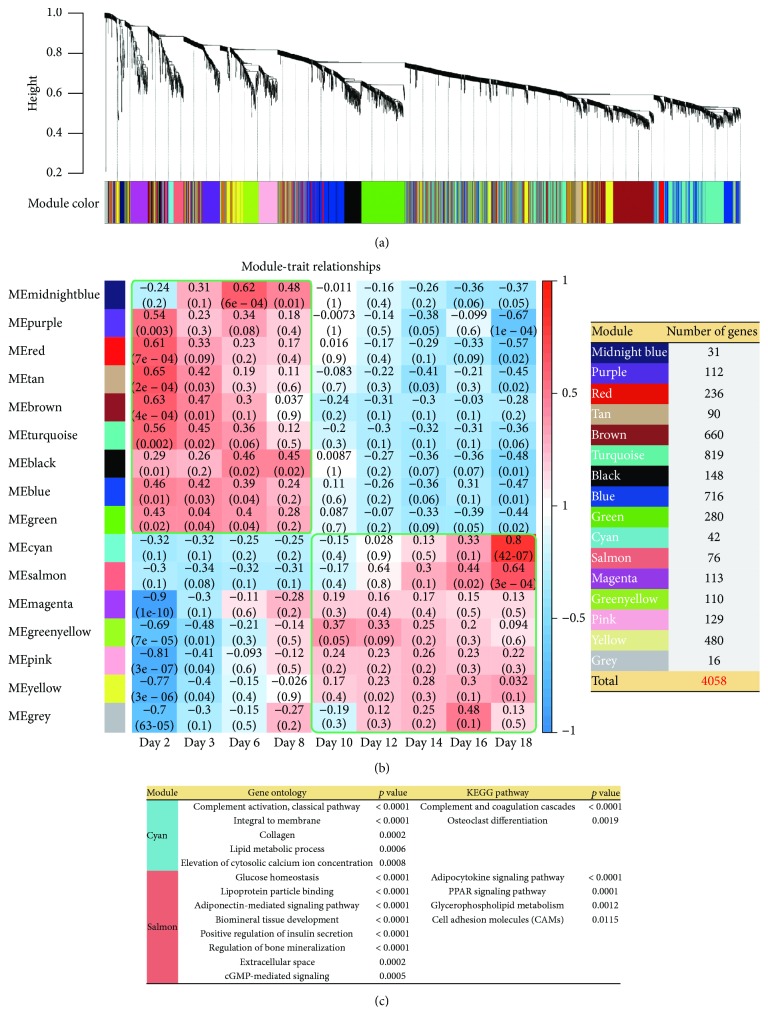
WGCNA analysis of 4058 time series gene expression data at the nine time points of osteoblast differentiation. (a) Hierarchical cluster trees depict coexpression modules identified using WGCNA. (b) A heat map plot of module-trait association. Each row corresponds to the module eigengene, the first principal component of a module. Each column corresponds to trait, the nine time points of osteoblast differentiation (days 2, 4, 6, 8, 10, 12, 14, 16, and 18). Each cell contains the corresponding correlation value and *p* value (left panel). Each of the sixteen modules contains between 16 and 819 genes (right panel). (c) Functional annotations of cyan and salmon modules. Gene ontology (GO) and KEGG pathway terms and corresponding *p* values are shown.

Gene coexpression patterns may provide information on gene networks or pathways related to different biological phenomena. We constructed a coexpression network of genes using time series gene expression data. The weighted gene coexpression network analysis (WGCNA) generated a total of 16 network modules (Figure 3(a)). The eigengenes for all 16 modules were calculated and the correlations between eigengenes and differentiation stages evaluated (Figure 3(b)). Genes belonging to 9 modules (midnight blue, purple, red, tan, brown, turquoise, black, blue, and green) were enriched with GO and KEGG pathways associated with the early osteoblast stage (days 2–8), such as “cell proliferation” and “osteoblast differentiation processes.” Meanwhile, genes belonging to the remaining 7 modules (cyan, salmon, magenta, green yellow, pink, yellow, and gray) were enriched with GO and KEGG pathways associated with the late osteoblast stage (days 10–18), such as “cell adhesion” and “osteoclast differentiation.”

Notably, the genes belonging to the cyan and salmon modules exhibited high trait association correlation (*r* > 0.6), and they were enriched with the following KEGG pathways and GO terms, besides “cell adhesion” and “osteoclast differentiation,” as mentioned above: PPAR signaling, subset of extracellular matrix, calcium metabolism, glucose homeostasis, cell signaling, and regulation of bone mineralization. This indicates that these two modules are strongly associated with late osteoblast differentiation (day 18). Thus, from the two modules (cyanide and salmon), we extracted 36 and 62 hub genes, respectively, with high connectivity (MM > 0.9), and these genes were used to find biomarkers for osteoblast differentiation as described below.

### 3.5. Identification of Markers Associated with Osteoblast Differentiation

We combined the results from gene clustering analysis and gene coexpression networks to determine novel biomarkers for osteoblast differentiation. Focusing on the late osteoblast differentiation stage, we considered genes belonging to clusters 1, 7, and 9 as well as the hub genes belonging to the cyan and salmon modules, as described above. We set the following cut-off values for biomarker candidates: absolute value of fold changes between early differentiation (average expression from samples of days 2–8) and late differentiation (average expression from samples of days 10–18) must be >2 and at least one of the samples should have an RPKM value > 5 for the gene.

We identified a total of 72 genes as potentially useful marker candidates for osteoblast differentiation (Supplementary Table 3). Functions associated with these genes include “regulation of bone mineralization (GO:0030500),” “extracellular matrix (GO:0031012),” “collagen (GO:0005581),” “collagen binding (GO:0005518),” and “cell adhesion (GO:0007155)” (Supplementary Table 4). Extracellular matrix, including collagen, is important for bone matrix construction [33], and cell adhesion affects cell-matrix interactions and further cell differentiation [34, 35].

**Figure 4:**
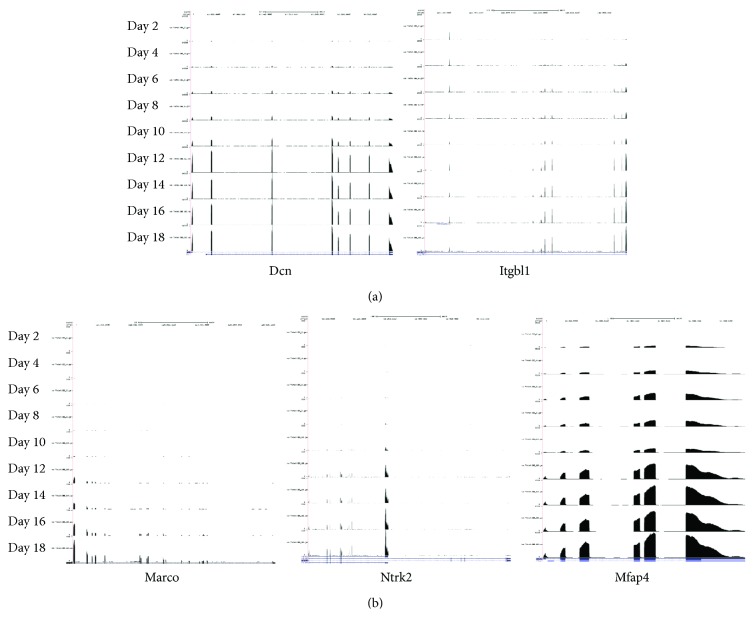
Expression patterns of five genes upregulated during late osteoblast differentiation stages. (a) Expression profiles of two known genes, Dcn (left panel) and Itgbl1 (right panel). (b) Expression profiles of three novel biomarker candidates, Marco (left panel), Ntrk2 (middle panel), Mfap4 (right panel).

Examples of genes associated with each function are as follows. Potential markers associated with “regulation of bone mineralization” are Ifitm5, Bglap, and Bglap2. Potential markers that are functionally associated with extracellular matrix included Abi3bp, Mfap4, Rarres2, Dcn, Dpt, Cilp, and Sod3 as well as the well-known gene Itgbl1 (integrin, beta-like 1; with EGF-like repeat domains) (Figure 4(a)). Dcn (decorin) is also known as a skeletal-related gene [36]. We identified C1qa, Marco, C1qc, Adipoq, and C1qb as collagen-related genes and identified Abi3bp, Srgn, and Dcn as genes related to “collagen binding.” Markers associated with “cell adhesion” included genes such as Cd36, Fbln7, Mfap4, Ibsp, and Dpt. In addition, Marco (macrophage receptor with collagen structure), Ntrk2 (neurotrophic tyrosine kinase receptor type 2), and Mfap4 (microfibrillar-associated protein 4) were identified as novel potential markers (Figure 4(b)). Although we identified potentially useful marker candidates for osteoblast differentiation, our study does have limitations. Since we rigorously filtered out 4058 time series genes based on the clustering analysis and WGCNA analysis (trait correlation and hub genes), the current method did not identify the known biomarkers (Fgfr2, Bmp4, and Fgf18) described in the clustering analysis. In addition, because the candidates were mainly identified based on the bioinformatic observations, their biological relevance would need to be further investigated at the cellular and molecular levels experimentally.

### 3.6. Expression Profiles of lncRNAs during Osteoblast Differentiation

**Figure 5:**
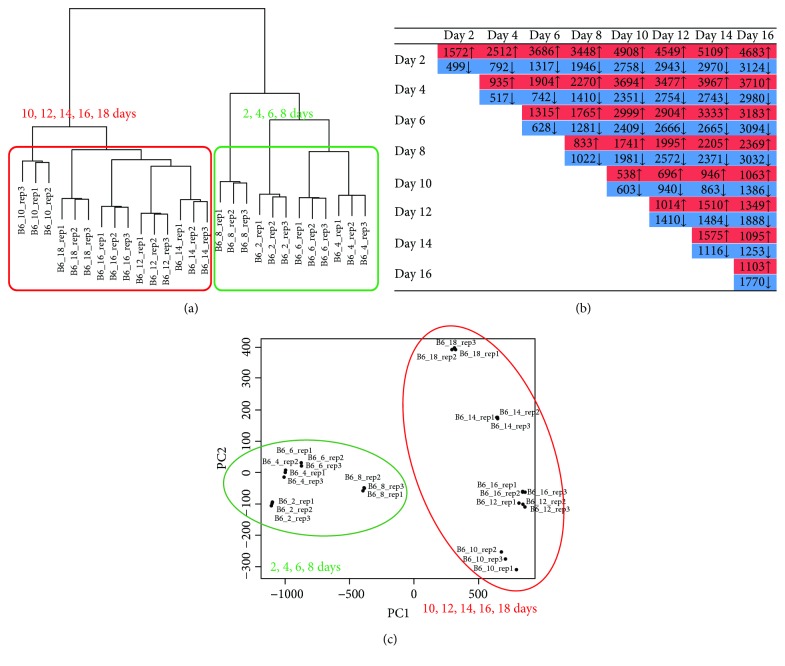
Global expression patterns of lncRNAs at the nine time points of osteoblast differentiation. (a) Hierarchical clustering of lncRNAs across the stages of osteoblast differentiation. (b) Number of lncRNAs showing up- or downregulation during osteoblast differentiation (fold change ≥ 2 or ≤0.5, FDR < 0.05). (c) Principal component analysis (PCA) of 3948 time series lncRNA expression profiles for different samples (three replicates for each time point of osteoblast differentiation).

The NONCODE (v4) database was used for lncRNA annotations, and at least one read was mapped onto 37,431 lncRNAs during osteoblast differentiation. Hierarchical clustering was performed based on the expression profiles in each sample. Upon application of similar approaches for known Ensembl genes to lncRNAs, we identified that 54% of the total lncRNAs (20,167 out of 37,431) were differentially expressed across the stages of osteoblast differentiation. Similar to the expression profile of mRNA described above, the expression patterns of lncRNAs were different between the early differentiation stage (2–8 days) and the late differentiation stage (10–18 days) (Figure 5(a)). The number of differentially expressed lncRNAs tended to increase with time (Figure 5(b)).

### 3.7. Dynamic Changes in lncRNAs during Osteoblast Differentiation

Examination of the temporal expression changes of lncRNAs revealed that levels of 3948 out of 37,431 lncRNAs were dynamically altered over the course of osteoblast differentiation. In PCA analysis using 3948 time series lncRNAs, 97% of the variations in lncRNA expression patterns could be explained by the first two principal components (PCs) (Figure 5(c)). Some of the 3948 time series lncRNAs highlighted were associated with osteogenic function such as “actin cytoskeleton organization,” “extracellular matrix organization,” “Wnt signaling pathway,” “embryonic skeletal system morphogenesis,” “Notch signaling pathway,” and “collagen fibril organization.”

### 3.8. Clustering Analysis of Time Series lncRNAs

**Figure 6:**
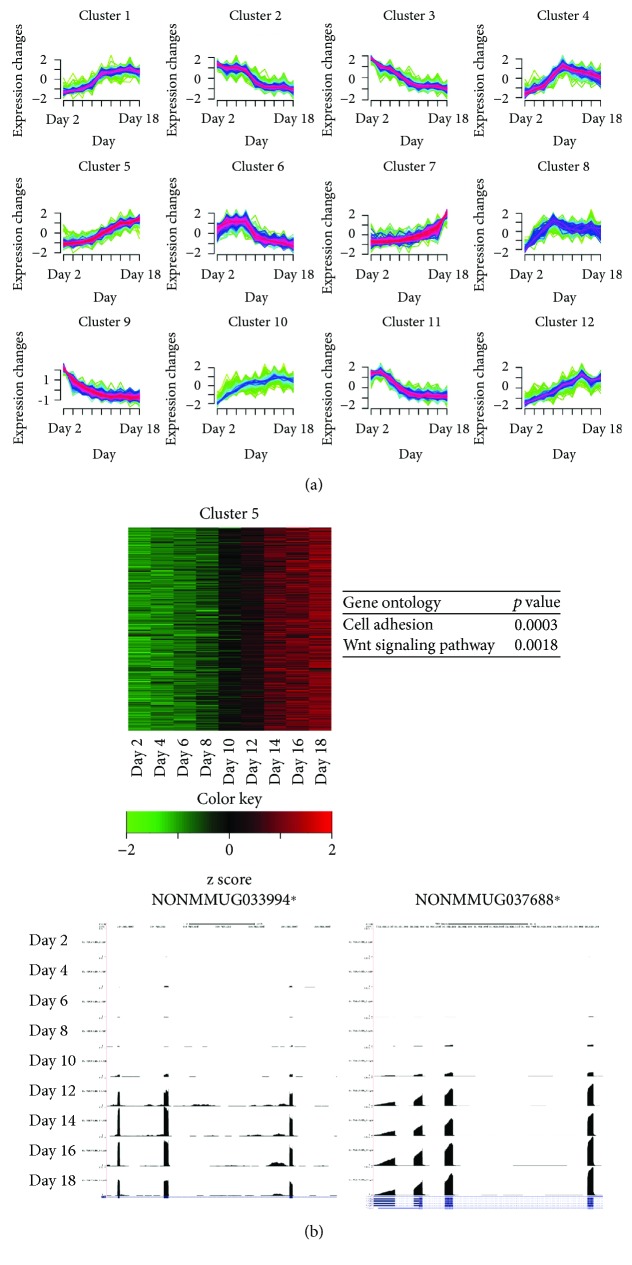
Clustering analysis of time series lncRNAs during osteoblast differentiation. (a) Soft clusters of 3948 time series lncRNAs generated by Mfuzz. The numbers on the *x*-axis (time, 1–9) correspond to the nine time points of osteoblast differentiation (days 2, 4, 6, 8, 10, 12, 14, 16, and 18). (b) Heat map of cluster 5. The color key indicates gene expression values (top left panel). Enriched GO terms and corresponding *p* values in cluster 5 are shown (top right panel). Gene expression profiles of two representative lncRNAs, NONMMUG033994 (bottom left panel) and NONMMUG037688 (bottom right panel), visualized in the bottom panel. ^∗^NONCODE v4 database ID.

Using R package Mfuzz [20], differentially expressed 3948 time series lncRNAs were divided into 12 clusters, and each cluster contained 142–595 lncRNAs (Figure 6(a)). The lncRNAs contained in 5 clusters (2, 3, 6, 9, and 11) were highly expressed in early osteoblast differentiation stage. Functional annotation analysis showed that these clusters are associated with “regulation of cell proliferation,” “actin cytoskeleton organization,” and “cell-cycle-related processes.” The remaining seven clusters (1, 4, 5, 7, 8, 10, and 12) included lncRNAs that displayed a higher expression in the late osteoblast differentiation stage and related to “cell adhesion,” “cell differentiation,” “collagen fibril organization,” “Wnt signaling pathway,” and “phosphorylation processes.”

### 3.9. Identification of lncRNAs Associated with Osteoblast Differentiation

Expression patterns of lncRNAs belonging to cluster 5 were not only very similar but also significantly increased over time, especially between 10 and 18 days. Thus, we focused on cluster 5 to find the lncRNAs markers associated with late differentiation of osteoblasts. Cut-off values were set for lncRNAs (Figure 6(b), upper panel), as in the procedures in novel biomarker identification for known genes.

In total, 89 lncRNAs were identified as potentially useful markers for osteoblast differentiation (Supplementary Table 5). GO enrichment analysis for these lncRNAs disclosed significant enrichment of “cell adhesion (GO:0007155)” and “Wnt signaling pathway (GO:0016055),” which is important in the differentiation and/or function of osteoblasts [31]. For examples, NONMMUG016555, NONMMUG038646, NONMMUG007704, NONMMUG001799, NONMMUG005875, NONMMUG002249, NONMMUG012067, NONMMUG035551, NONMMUG015487, and NONMMUG035553 are related to “cell adhesion.” NONMMUG038646, NONMMUG016590, and NONMMUG001799 are related to “Wnt signaling pathway (GO:0016055).” In addition, we identified novel candidate lncRNA markers, and two examples are presented in Figure 6(b) (bottom panel).

Next, to see whether the identified lncRNAs could be regulated by common regulation factors, we searched the sequence motifs in the promoter regions (the upstream 1000 bp nucleotide sequences of lncRNAs) of the 89 lncRNAs with background controls generated from all NONCODE lncRNA promoter regions. We performed the motif enrichment analysis using MEME Suite package [23] and the HOCOMOCO v11 mouse transcription factor database [24] to detect known transcription factor binding motifs that are significantly enriched within the promoter region. A total of five significant motifs were identified (Table 3). The most significant enriched motif (WT1_MOUSE.H11MO.1.A) was the Wilms tumor protein (Wt1) transcription factor binding motif. Recently, it was shown that Wt1 was expressed during development in the limb tissue of E11.5 to E16.5 mice and the loss of the Wt1 expression affected a reduction of non-haematopoetic MSC cells in the E18.5 hindlimb [37]. These findings suggest that Wt1 may play a functional role in the bone development. The second most significant enriched motif (EGR2_MOUSE.H11MO.0.A) was early growth response 2 (Egr2) transcription factor binding motif. Chandra et al. previously suggested that EGFR-induced Egr2 expression is crucial for osteoprogenitor maintenance and new bone formation [38]. Further research on the multiple layers of regulatory mechanisms, including lncRNA expression and transcription regulations, will be required to fully understand the role of these lncRNAs during bone development.

## 4. Conclusions

We employed public RNA-Seq data (NCBI GEO accession number GSE54461) to profile mRNA and lncRNA expression and identified a series of biomarkers potentially associated with bone differentiation. Data from the current study showed that a combination of bioinformatic approaches, including (1) time series, (2) clustering, and (3) coexpression network analyses, provides an effective means to identify novel candidate markers associated with osteoblast differentiation. Expression patterns of mRNA and lncRNA during osteoblast differentiation were defined as two distinct stages (early and late osteoblast differentiation). Functional annotation showed that the members in the late stage are involved in osteogenesis processes. Notably, common transcription factor binding motifs were enriched in the identified lncRNA markers. Our findings provide a valuable resource for lncRNA study and understanding of the mechanism of mouse osteoblast differentiation. Further research is needed to determine the functions of lncRNA and mRNA identified as potential markers in this study.

## Figures and Tables

**Table 1:** RNA-Seq read mapping summary.

SRA ID	Sample	Total reads	Uniquely mapped reads	% of uniquely mapped reads
SRR1146385	B6_2_rep1	20,605,665	18,353,730	89.07
SRR1146386	B6_2_rep2	21,204,511	18,881,462	89.04
SRR1146387	B6_2_rep3	21,196,674	18,867,579	89.01
SRR1146388	B6_4_rep1	21,042,912	18,976,744	90.18
SRR1146389	B6_4_rep2	21,636,813	19,507,642	90.16
SRR1146390	B6_4_rep3	21,628,526	19,496,233	90.14
SRR1146391	B6_6_rep1	22,012,993	19,750,118	89.72
SRR1146392	B6_6_rep2	22,652,935	20,316,973	89.69
SRR1146393	B6_6_rep3	22,656,580	20,317,497	89.68
SRR1146394	B6_8_rep1	16,068,544	14,395,620	89.59
SRR1146395	B6_8_rep2	16,509,407	14,785,805	89.56
SRR1146396	B6_8_rep3	16,498,155	14,769,672	89.52
SRR1146397	B6_10_rep1	11,685,016	10,532,491	90.14
SRR1146398	B6_10_rep2	12,016,157	10,826,863	90.10
SRR1146399	B6_10_rep3	12,016,476	10,827,771	90.11
SRR1146400	B6_12_rep1	32,109,724	29,035,678	90.43
SRR1146401	B6_12_rep2	33,017,225	29,851,897	90.41
SRR1146402	B6_12_rep3	32,970,446	29,805,326	90.40
SRR1146403	B6_14_rep1	22,996,425	20,867,104	90.74
SRR1146404	B6_14_rep2	23,630,602	21,437,833	90.72
SRR1146405	B6_14_rep3	23,616,942	21,422,044	90.71
SRR1146406	B6_16_rep1	26,956,212	24,189,458	89.74
SRR1146407	B6_16_rep2	27,696,210	24,846,901	89.71
SRR1146408	B6_16_rep3	27,659,416	24,807,073	89.69
SRR1146409	B6_18_rep1	22,811,796	20,549,338	90.08
SRR1146410	B6_18_rep2	23,478,940	21,148,934	90.08
SRR1146411	B6_18_rep3	23,452,037	21,114,152	90.03

**Table 2:** Enriched GO terms and KEGG pathways of cluster 1, 7, and 9 genes.

Cluster	Gene ontology	*p* value	KEGG pathway	*p* value
Cluster 1	Wnt-activated receptor activity	<0.0001	ECM-receptor interactions	0.0007
	Collagen type I	<0.0001	Focal adhesion	0.0022
	Calcium ion binding	0.0001		
	Osteoblast differentiation	0.0003		
	Wnt-protein binding	0.0003		
Cluster 7	Collagen binding	<0.0001	Cytokine-cytokine receptor interactions	0.004
	Positive regulation of cell-substrate adhesion	<0.0001		
	Extracellular region	<0.0001		
	Extracellular matrix	<0.0001		
	Positive regulation of osteoblast differentiation	0.0001		
Cluster 9	Collagen	<0.0001	PPAR signaling pathway	<0.0001
	Biomineral tissue development	<0.0001	Osteoclast differentiation	0.001
	Regulation of bone mineralization	<0.0001	Adipocytokine signaling pathway	0.006
	Extracellular region	<0.0001	Cytokine-cytokine receptor interactions	0.0089
	Extracellular space	<0.0001		
Cluster 10	Extracellular matrix	<0.0001		

**Table 3:** Enriched transcriptional binding motifs in the 89 lncRNA promoter regions.

Motif ID	Transcription factor	*p* value	Adjusted *p* value
WT1_MOUSE.H11MO.1.A	Wt1	8.014E-06	0.004246
EGR2_MOUSE.H11MO.0.A	Egr2	5.517E-05	0.02887
ZN281_MOUSE.H11MO.0.A	Zn281	5.737E-05	0.03001
ZIC1_MOUSE.H11MO.0.B	Zic1	7.868E-05	0.04092
SP5_MOUSE.H11MO.1.C	Sp5	9.138E-05	0.04737

## Supplementary Materials

Supplementary Materials Supplementary Table 1: functional annotation of upregulated genes on day 18 (day 2 versus day 18). Supplementary Table 2: functional annotation of downregulated genes on day 18 (day 2 versus day 18). Supplementary Table 3: known Ensembl genes potentially associated with osteoblast differentiation. Supplementary Table 4: enriched GO terms of known Ensembl genes potentially associated with osteoblast differentiation. Supplementary Table 5: lncRNAs (NONCODE v4) potentially associated with osteoblast differentiation. Supplementary Figure 1: Overview of the methods and procedures to identify biomarkers. Supplementary Figure 2: proportion of variance for principal components. Supplementary Figure 3: expression profiles of known mature osteoblast markers.
