# Supplementary material for: Differential Expression Profiling of Long Noncoding RNA and mRNA during Osteoblast Differentiation in Mouse
Source: Int J Genomics. 2018 Mar 22;2018:7691794. doi: 10.1155/2018/7691794 (PMC5885395; doi:10.1155/2018/7691794)

# SUPPLEMENTARY METHODS

## Time series analysis of differential gene expression

R package DESeq [1] and EdgeR [2] were employed to identify genes that were differentially expressed across the differentiation time-period to a significant extent, designated time series genes. We selected time series genes that displayed significant differential expression with FDR <5% in both DESeq and EdgeR, absolute fold change $\geq$2 (between day 2 and at least one other time-point), and maximum RPKM $\geq$3 across the time series. Details of the methods for each package are described in the below.

DESeq procedure: Gene dispersion was calculated using the ‘pooled Cox-Reid’ method [1]. We employed two generalized linear models (GLM). The first was fitted based on ‘time’ and ‘replicate identifier’ factors while the second was fitted using only the ‘replicate identifier’ factor. The two models were compared to establish whether the time factor exerted a significant effect on gene expression. The Benjamini-Hochberg procedure was applied for multiple testing corrections.

EdgeR procedure: Gene dispersion was calculated using the Cox-Reid method [2]. A design matrix containing ‘time’ and ‘replicate identifier’ factors was created for estimating dispersion. A generalized linear model (GLM) was fitted for each gene and a likelihood ratio test performed, which included all coefficients from the design matrix. The above method facilitated the identification of genes that were significantly altered across the time-course of osteoblast differentiation. The Benjamini-Hochberg procedure was applied for multiple testing corrections.

## Weighted gene co-expression network analysis

Gene co-expression network analysis was performed using the R package 'WGCNA' [3]. We employed weighted gene co-expression network analysis using the RPKM values of the time series genes that were differentially expressed to a significant extent across the time-points in both DESeq and EdgeR. A weighted correlation network was constructed by creating a matrix of pairwise

correlations between all samples. The soft-thresholding power β = 20 was selected to obtain an adjacency matrix. To identify the network modules of co-expressed genes, we constructed a topological overlap-based dissimilarity matrix. Genes with highly similar co-expression relationships were grouped based on hierarchical clustering on the topological overlap. We used the Dynamic Tree Cut algorithm to cut the hierarchal clustering tree and define modules (minModuleSize: 30). Modules were tested for their associations with the trait by correlating module eigengenes with the time-point trait (collected time-points: 1 and 0). Genes within modules that tend to display high network connectivity are designated 'hub genes'. To define hub genes, we used Module membership measurement (MM, also known as module eigengene-based connectivity, kME) that explains a measure of the strength of membership of a gene in a module obtained from WGCNA analysis. In cases where the kME value of gene in a specific module was greater than 0.9, the gene was defined as a 'hub gene' within a module.

**Gene ontology (GO) term and KEGG pathway enrichment analysis**

GO terms of each gene were obtained from Ensembl BioMart and KEGG pathways from the KEGG PATHWAY database. To analyze GO category or KEGG pathway enrichment in a specific gene set, the fraction of genes in the test set (Ftest) associated with each GO category was calculated. Next, we generated the random control gene set with the same number of genes as the test set. In this procedure, the random control gene was selected by matching the length of the test gene. The fraction of genes in the randomly selected control set (Fcontrol) associated with the current GO category or KEGG pathway was calculated. This random sampling process was repeated 10,000 times. A p-value cutoff (1/total number of GO terms considered) was set to select significantly enriched GO terms or KEGG pathways.

**Analysis of motifs enrichment**

AME (analysis of motif enrichment) module in the MEME Suite 4.12.20 [4] was used for the

identification of motifs in the promoter regions on the lncRNAs identified as potential markers. Basically, AME trys to identify known motifs (in the various databases) that are relatively enriched in the sequences compared with the control sequences. For the promoter regions, we extracted the upstream 1,000bp nucleotides from the transcription starting sites of the 89 lncRNAs. To generate the control sequences, we also extracted the same length of the promoter sequences from the all NOCODE lncRNAs. HOmo sapiens COmprehensive MOdel COllection (HOCOMOCO) v11 Mouse transcription factor database [5] was used. Other parameters were set as defaults suggested by the guidelines in the MEME Suite. Adjusted P-value of 0.05 was used for the statistical significance.

## References


[1] Anders S, Huber W, Differential expression analysis for sequence count data. Genome Biol. 2010;11(10):R106.

[2] Robinson MD, McCarthy DJ, Smyth GK, edgeR: a Bioconductor package for differential expression analysis of digital gene expression data. Bioinformatics. 2010 Jan 1;26(1):139-40.

[3] Langfelder P, Horvath S, WGCNA: an R package for weighted correlation network analysis. BMC Bioinformatics. 2008 Dec 29;9:559.

[4] McLeay RC1, Bailey TL, Motif Enrichment Analysis: a unified framework and an evaluation on ChIP data. MC Bioinformatics. 2010 Apr 1;11:165.

[5] Kulakovskiy IV, Vorontsov IE, Yevshin IS, Sharipov RN, Fedorova AD, Rumynskiy EI, Medvedeva YA, Magana-Mora A, Bajic VB, Papatsenko DA, Kolpakov FA, Makeev VJ. HOCOMOCO: towards a complete collection of transcription factor binding models for human and mouse via large-scale ChIP-Seq analysis. Nucleic Acids Res. 2017 Nov 11.

**SUPPLEMENTARY TABLES AND FIGURES**

**Supplementary Table 1. Functional annotation of upregulated genes on Day18 (Day 2 vs Day 18)**

| **Gene ontology** | **P-value** | **KEGG pathway** | **P-value** |
|---|---|---|---|
| Extracellular matrix binding | <0.0001 | Cytokine-cytokine receptor interaction | <0.0001 |
| Interstitial matrix | <0.0001 | Cell adhesion molecules (CAMs) | <0.0001 |
| Positive regulation of non-canonical Wnt receptor signaling pathway | <0.0001 | ECM-receptor interaction | <0.0001 |
| Positive regulation of bone mineralization | <0.0001 | Calcium signaling pathway | <0.0001 |
| Collagen type I | <0.0001 | PPAR signaling pathway | <0.0001 |
| Calcium- and calmodulin-responsive Adenylate cyclase activity | <0.0001 | Osteoclast differentiation | <0.0001 |
| Extracellular space | <0.0001 | Chemokine signaling pathway | 0.001 |
| BMP receptor binding | <0.0001 | Focal adhesion | 0.0011 |
| Collagen type IX | <0.0001 | | |
| Extracellular matrix organization | <0.0001 | | |
| BMP signaling pathway involved in Heart induction | <0.0001 | | |
| Wnt-protein binding | <0.0001 | | |
| Fibrillar collagen | <0.0001 | | |
| Cell adhesion | <0.0001 | | |
| Elevation of cytosolic calcium ion concentration | <0.0001 | | |
| Ossification | <0.0001 | | |
| Integral to membrane | <0.0001 | | |
| Positive regulation of insulin-like growth factor receptor signaling pathway | <0.0001 | | |

| | | | |
|---|---|---|---|
| Wnt-activated receptor activity | <0.0001 | | |
| Positive regulation of tooth mineralization | <0.0001 | | |
| Negative regulation of biomineral tissue development | <0.0001 | | |
| Collagen catabolic process | <0.0001 | | |
| Bone mineralization involved in bone maturation | <0.0001 | | |
| Calcium ion binding | <0.0001 | | |
| Regulation of cytokine secretion | 0.0001 | | |
| Positive regulation of Wnt receptor signaling pathway | 0.0001 | | |
| Odontogenesis of dentin-containing tooth | 0.0001 | | |

**Supplementary Table 2. Functional annotation of downregulated genes on Day18 (Day 2 vs Day18)**

| **Gene ontology** | **P-value** | **KEGG pathway** | **P-value** |
|---|---|---|---|
| Cell proliferation | <0.0001 | Cell cycle | <0.0001 |
| Cell division | <0.0001 | RNA degradation | <0.0001 |
| Regulation of attachment of spindle microtubules to kinetochore | <0.0001 | DNA replication | <0.0001 |
| Muscle thin filament tropomyosin | <0.0001 | Cytokine-cytokine receptor interaction | 0.0004 |
| G1/S transition of mitotic cell cycle | <0.0001 | RNA polymerase | 0.0004 |
| Regulation of cell cycle | <0.0001 | Adherens junction | 0.0029 |
| M phase of mitotic cell cycle | <0.0001 | | |
| Mitotic spindle organization | <0.0001 | | |
| Chromosome segregation | <0.0001 | | |

| | | |
|---|---|---|
| G2/M transition of mitotic cell cycle | <0.0001 | |
| Condensed chromosome outer kinetochore | <0.0001 | |
| Condensed chromosome | <0.0001 | |
| Translation | <0.0001 | |
| Microtubule binding | <0.0001 | |
| DNA replication | <0.0001 | |
| Structural constituent of ribosome | <0.0001 | |
| DNA replication initiation | <0.0001 | |
| DNA replication, removal of RNA primer | <0.0001 | |
| DNA repair | <0.0001 | |
| Positive regulation of cell proliferation | 0.0001 | |
| Response to DNA damage stimulus | 0.0001 | |
| Negative regulation of RNA splicing | 0.0001 | |

**Supplementary Table 3. Known Ensembl genes potentially associated with osteoblast differentiation**

| Ensembl gene id | Gene symbol | Description | Cluster | Module |
|---|---|---|---|---|
| ENSMUSG00000061780 | Cfd | complement factor D (adipsin) | cl.9 | salmon |
| ENSMUSG00000030278 | Cidec | cell death-inducing DFFA-like effector c | cl.9 | salmon |
| ENSMUSG00000022878 | Adipoq | adiponectin, C1Q and collagen domain containing | cl.9 | salmon |
| ENSMUSG00000027559 | Car3 | carbonic anhydrase 3 | cl.9 | salmon |
| ENSMUSG00000074483 | Bglap | bone gamma carboxyglutamate protein | cl.9 | salmon |
| ENSMUSG00000029306 | Ibsp | integrin binding sialoprotein | cl.9 | salmon |
| ENSMUSG00000030546 | Plin1 | perilipin 1 | cl.9 | salmon |
| ENSMUSG00000072553 | Gm525 | protein coding gene | cl.9 | salmon |
| ENSMUSG00000023019 | Gpd1 | glycerol-3-phosphate dehydrogenase 1 (soluble) | cl.9 | salmon |
| ENSMUSG00000069515 | Lyz1 | lysozyme 1 | cl.9 | salmon |
| ENSMUSG00000021322 | Aoah | acyloxyacyl hydrolase | cl.9 | cyan |
| ENSMUSG00000089671 | Gm16537 | antisense lncRNA gene | cl.9 | salmon |
| ENSMUSG00000074486 | Bglap2 | bone gamma-carboxyglutamate protein 2 | cl.9 | salmon |
| ENSMUSG00000026390 | Marco | macrophage receptor with collagenous structure | cl.9 | cyan |
| ENSMUSG00000035686 | Thrsp | thyroid hormone responsive | cl.9 | cyan |
| ENSMUSG00000050711 | Scg2 | secretogranin II | cl.9 | salmon |
| ENSMUSG00000039084 | Chad | chondroadherin | cl.9 | cyan |

| | | | | |
|---|---|---|---|---|
| ENSMUSG00000002944 | Cd36 | CD36 antigen | cl.9 | salmon |
| ENSMUSG00000086868 | Gm15883 | antisense lncRNA gene | cl.9 | cyan |
| ENSMUSG00000052974 | Cyp2f2 | cytochrome P450, family 2, subfamily f, polypeptide 2 | cl.9 | cyan |
| ENSMUSG00000030579 | Tyrobp | TYRO protein tyrosine kinase binding protein | cl.9 | salmon |
| ENSMUSG00000028581 | Laptm5 | lysosomal-associated protein transmembrane 5 | cl.9 | cyan |
| ENSMUSG00000036896 | C1qc | complement component 1, q subcomponent, C chain | cl.9 | cyan |
| ENSMUSG00000004730 | Emr1 | Adgre1, adhesion G protein-coupled receptor E1 | cl.9 | cyan |
| ENSMUSG00000042254 | Cilp | cartilage intermediate layer protein, nucleotide pyrophosphohydrolase | cl.9 | salmon |
| ENSMUSG00000007682 | Dio2 | deiodinase, iodothyronine, type II | cl.9 | salmon |
| ENSMUSG00000036887 | C1qa | complement component 1, q subcomponent, alpha polypeptide | cl.9 | cyan |
| ENSMUSG00000058715 | Fcer1g | Fc receptor, IgE, high affinity I, gamma polypeptide | cl.9 | salmon |
| ENSMUSG00000013584 | Aldh1a2 | aldehyde dehydrogenase family 1, subfamily A2 | cl.9 | salmon |
| ENSMUSG00000026712 | Mrc1 | mannose receptor, C type 1 | cl.9 | cyan |
| ENSMUSG00000036905 | C1qb | complement component 1, q subcomponent, beta polypeptide | cl.9 | cyan |
| ENSMUSG00000030144 | Clec4d | C-type lectin domain family 4, member d | cl.9 | cyan |
| ENSMUSG00000062593 | Lilrb4 | leukocyte immunoglobulin-like receptor, subfamily B, member 4A | cl.9 | cyan |
| ENSMUSG00000027386 | Fbln7 | fibulin 7 | cl.9 | salmon |
| ENSMUSG00000055254 | Ntrk2 | neurotrophic tyrosine kinase, receptor, type 2 | cl.9 | salmon |
| ENSMUSG00000069516 | Lyz2 | lysozyme 2 | cl.9 | salmon |

| | | | | |
|---|---|---|---|---|
| ENSMUSG00000021998 | Lcp1 | lymphocyte cytosolic protein 1 | cl.9 | cyan |
| ENSMUSG00000049130 | C5ar1 | complement component 5a receptor 1 | cl.9 | cyan |
| ENSMUSG00000038642 | Ctss | cathepsin S | cl.9 | cyan |
| ENSMUSG00000020077 | Srgn | serglycin | cl.9 | salmon |
| ENSMUSG00000046805 | Mpeg1 | Macrophage Expressed 1 | cl.9 | cyan |
| ENSMUSG00000002985 | Apoe | Apolipoprotein E | cl.9 | salmon |
| ENSMUSG00000021319 | Sfrp4 | Secreted Frizzled-Related Protein 4 | cl.9 | salmon |
| ENSMUSG00000018774 | Cd68 | CD68 Molecule | cl.9 | cyan |
| ENSMUSG00000009281 | Rarres2 | retinoic acid receptor responder (tazarotene induced) 2 | cl.9 | salmon |
| ENSMUSG00000033715 | Akr1c14 | aldo-keto reductase family 1, member C14 | cl.9 | salmon |
| ENSMUSG00000022425 | Enpp2 | Ectonucleotide Pyrophosphatase/Phosphodiesterase 2 | cl.9 | salmon |
| ENSMUSG00000042684 | Npl | N-acetylneuraminate pyruvate lyase (dihydrodipicolinate synthase) | cl.9 | cyan |
| ENSMUSG00000018796 | Acsl1 | Acyl-CoA Synthetase Long-Chain Family Member 1 | cl.9 | salmon |
| ENSMUSG00000030747 | Dgat2 | diacylglycerol O-acyltransferase 2 | cl.9 | salmon |
| ENSMUSG00000053714 | 4732471J01Rik | RIKEN cDNA 4732471J01 gene | cl.9 | salmon |
| ENSMUSG00000025489 | Ifitm5 | Interferon Induced Transmembrane Protein 5 | cl.9 | salmon |
| ENSMUSG00000040552 | C3ar1 | Complement Component 3a Receptor 1 | cl.9 | cyan |
| ENSMUSG00000042379 | Esm1 | endothelial cell-specific molecule 1 | cl.9 | salmon |
| ENSMUSG00000025509 | Pnpla2 | Patatin-Like Phospholipase Domain Containing 2 | cl.9 | salmon |

| | | | | |
|---|---|---|---|---|
| ENSMUSG00000024810 | Il33 | Interleukin 33 | cl.7 | yellow |
| ENSMUSG00000078169 | Gm16499 | predicted gene 16499 | cl.7 | yellow |
| ENSMUSG00000019929 | Dcn | Decorin | cl.7 | yellow |
| ENSMUSG00000024621 | Csf1r | Colony Stimulating Factor 1 Receptor | cl.9 | cyan |
| ENSMUSG00000039899 | Fgl2 | Fibrinogen-Like 2 | cl.9 | salmon |
| ENSMUSG00000054404 | Slfn5 | Schlafen Family Member 5 | cl.7 | yellow |
| ENSMUSG00000074776 | Gm10754 | predicted gene 10754 | cl.7 | yellow |
| ENSMUSG00000035258 | Abi3bp | ABI Family, Member 3 (NESH) Binding Protein | cl.7 | yellow |
| ENSMUSG00000044986 | Tst | Thiosulfate Sulfurtransferase (Rhodanese) | cl.9 | salmon |
| ENSMUSG00000061353 | Cxcl12 | Chemokine (C-X-C Motif) Ligand 12 | cl.7 | yellow |
| ENSMUSG00000089961 | Gm16567 | predicted gene 16567 | cl.7 | yellow |
| ENSMUSG00000038521 | C1s | complement component 1, s subcomponent | cl.7 | yellow |
| ENSMUSG00000070509 | Rgma | Repulsive Guidance Molecule Family Member A | cl.7 | yellow |
| ENSMUSG00000032925 | Itgbl1 | integrin, beta-like 1 (with EGF-like repeat domains) | cl.7 | yellow |
| ENSMUSG00000042436 | Mfap4 | microfibrillar-associated protein 4 | cl.9 | yellow |
| ENSMUSG00000026574 | Dpt | Dermatopontin | cl.9 | yellow |
| ENSMUSG00000072941 | Sod3 | superoxide dismutase 3, extracellular | cl.7 | yellow |

**Supplementary Table 4. Enriched GO terms of known Ensembl genes potentially associated with osteoblast differentiation**

| **Gene ontology** | **P-value** | **Genes** |
|---|---|---|
| Regulation of bone mineralization | <0.0001 | Ifitm5, Bglap, Bglap2 |
| Extracellular matrix | <0.0001 | Abi3bp, Mfap4, Rarres2, Dcn, Dpt, Cilp, Sod3 |
| Collagen | <0.0001 | C1qa, Marco, C1qc, Adipoq, C1qb |
| Collagen binding | 0.0001 | Abi3bp, Srgn, Dcn |
| Extracellular region | 0.0002 | Esm1, C1s, Lyz1, Lyz2, Cxcl12, Aoah, Itgbl1, Apoe, Bglap, Rarres2, Ibsp, Fgl2, Gm525, Bglap2 |
| Cell adhesion | 0.0009 | Cd36, Fbln7, Mfap4, Ibsp, Dpt |

**Supplementary Table 5. LncRNAs (NONCODE v4) potentially associated with osteoblast differentiation**

| LncRNA (noncode v4) | Position (Chr\|start\|end\|) | GO terms | Cluster |
|---|---|---|---|
| NONMMUG040772 | chr8\|68880555\|68889681\| | NA | cl.5 |
| NONMMUG002249 | chr1\|162648229\|162649996\| | GO:0008150\|GO:0016311\|GO:0007010\|GO:0006810\|GO:0030036\|GO:0009117\|GO:0046079\|GO:0007605\|GO:0008584\|GO:0007155\| | cl.5 |
| NONMMUG040773 | chr8\|68892362\|68893093\| | GO:0008150\|GO:0006810\|GO:0006355\|GO:0007275\|GO:0006351\|GO:0055114\|GO:0016310\|GO:0060048\|GO:0007507\|GO:0006811\| | cl.5 |
| NONMMUG001261 | chr1\|87264481\|87265174\| | NA | cl.5 |
| NONMMUG037688 | chr7\|31051681\|31053122\| | NA | cl.5 |
| NONMMUG038418 | chr7\|78302323\|78377822\| | NA | cl.5 |
| NONMMUG033994 | chr5\|134725603\|134729106\| | NA | cl.5 |
| NONMMUG028069 | chr3\|138281408\|138307022\| | GO:0008150\|GO:0055114\|GO:0008152\|GO:0006810\|GO:0006629\|GO:0008203\|GO:0006508\|GO:0008202\|GO:0007596\|GO:0006631\| | cl.5 |
| NONMMUG044832 | chrX\|48108921\|48112154\| | NA | cl.5 |
| NONMMUG014052 | chr15\|10213465\|10319278\| | NA | cl.5 |
| NONMMUG001957 | chr1\|143640769\|143641091\| | NA | cl.5 |
| NONMMUG034057 | chr5\|136742755\|136747701\| | NA | cl.5 |
| NONMMUG013244 | chr14\|65968654\|65972537\| | NA | cl.5 |

| | | | |
|---|---|---|---|
| NONMMUG014054 | chr15\|10333361\|10337011\| | NA | cl.5 |
| NONMMUG019603 | chr18\|33461141\|33463623\| | NA | cl.5 |
| NONMMUG001262 | chr1\|87309226\|87309742\| | NA | cl.5 |
| NONMMUG013545 | chr14\|79637816\|79645658\| | NA | cl.5 |
| NONMMUG021052 | chr19\|24678261\|24678926\| | NA | cl.5 |
| NONMMUG026964 | chr3\|83773853\|83789956\| | NA | cl.5 |
| NONMMUG021053 | chr19\|24679681\|24682233\| | NA | cl.5 |
| NONMMUG004738 | chr10\|115520533\|115587743\| | NA | cl.5 |
| NONMMUG013240 | chr14\|65763206\|65765489\| | NA | cl.5 |
| NONMMUG035552 | chr6\|65704336\|65706790\| | NA | cl.5 |
| NONMMUG013934 | chr15\|3270999\|3275122\| | NA | cl.5 |
| NONMMUG035551 | chr6\|65703417\|65704607\| | GO:0008150\|GO:0007275\|GO:0007165\|GO:0006355\|GO:0006810\|GO:0030154\|GO:0008152\|GO:0007155\|GO:0006351\|GO:0045944\| | cl.5 |
| NONMMUG004735 | chr10\|115452532\|115453337\| | NA | cl.5 |
| NONMMUG025750 | chr2\|174281237\|174295436\| | NA | cl.5 |
| NONMMUG013935 | chr15\|3280617\|3284213\| | GO:0055114\|GO:0008150\|GO:0032092\|GO:0051017\|GO:0006940\|GO:0006629\|GO:0042593\|GO:0006694\|GO:0008202\|GO:0008203\| | cl.5 |

| | | | |
|---|---|---|---|
| NONMMUG004736 | chr10\|115456648\|115463079\| | NA | cl.5 |
| NONMMUG007968 | chr11\|110026848\|110030081\| | NA | cl.5 |
| NONMMUG042503 | chr9\|37544954\|37546138\| | NA | cl.5 |
| NONMMUG023159 | chr2\|62147862\|62158283\| | NA | cl.5 |
| NONMMUG035553 | chr6\|65709768\|65712321\| | GO:0008150\|GO:0007275\|GO:0007165\|GO:0006355\|GO:0006810\|GO:0030154\|GO:0006351\|GO:0008152\|GO:0007155\|GO:0006811\| | cl.5 |
| NONMMUG028340 | chr3\|157313650\|157316442\| | NA | cl.5 |
| NONMMUG023171 | chr2\|62527349\|62563717\| | NA | cl.5 |
| NONMMUG023648 | chr2\|84773131\|84775128\| | NA | cl.5 |
| NONMMUG023162 | chr2\|62317390\|62325377\| | NA | cl.5 |
| NONMMUG046342 | chrX\|167317680\|167330443\| | NA | cl.5 |
| NONMMUG007553 | chr11\|99042186\|99048360\| | NA | cl.5 |
| NONMMUG046324 | chrX\|166344842\|166373287\| | NA | cl.5 |
| NONMMUG028820 | chr4\|33173284\|33174835\| | NA | cl.5 |

| | | | |
|---|---|---|---|
| NONMMUG039466 | chr7\|132154171\|132155301\| | GO:0008150\|GO:0007165\|GO:0006355\|GO:0006351\|GO:0006955\|GO:0019886\|GO:0007186\|GO:0045893\|GO:0045087\|GO:0045944\| | cl.5 |
| NONMMUG027238 | chr3\|94933159\|94938075\| | NA | cl.5 |
| NONMMUG029346 | chr4\|57063030\|57070559\| | NA | cl.5 |
| NONMMUG035956 | chr6\|88843538\|88845925\| | NA | cl.5 |
| NONMMUG009430 | chr12\|81185824\|81186410\| | NA | cl.5 |
| NONMMUG024856 | chr2\|139677188\|139680259\| | NA | cl.5 |
| NONMMUG014545 | chr15\|55252556\|55259273\| | NA | cl.5 |
| NONMMUG037022 | chr6\|148232462\|148236189\| | NA | cl.5 |
| NONMMUG004516 | chr10\|97479632\|97481289\| | NA | cl.5 |
| NONMMUG038646 | chr7\|89410170\|89413127\| | GO:0008150\|GO:0007275\|GO:0007165\|GO:0016055\|GO:0006355\|GO:0006351\|GO:0030335\|GO:0007155\|GO:0043066\|GO:0016477\| | cl.5 |
| NONMMUG015285 | chr15\|92771037\|92771819\| | NA | cl.5 |
| NONMMUG046268 | chrX\|162546220\|162552579\| | NA | cl.5 |
| NONMMUG013995 | chr15\|7193513\|7195875\| | NA | cl.5 |
| NONMMUG003936 | chr10\|70284235\|70284953\| | NA | cl.5 |
| NONMMUG042782 | chr9\|51851558\|51853855\| | NA | cl.5 |
| NONMMUG045434 | chrX\|98320098\|98323212\| | NA | cl.5 |

| | | | |
|---|---|---|---|
| NONMMUG016557 | chr16\|43640086\|43642602\| | NA | cl.5 |
| NONMMUG007554 | chr11\|99051575\|99052641\| | NA | cl.5 |
| NONMMUG020747 | chr19\|7586617\|7587918\| | NA | cl.5 |
| NONMMUG004220 | chr10\|82651308\|82653199\| | NA | cl.5 |
| NONMMUG014605 | chr15\|58941244\|58942061\| | NA | cl.5 |
| NONMMUG001799 | chr1\|133906531\|133914822\| | GO:0008150\|GO:0007155\|GO:0016055\|GO:0007275\|GO:0006355\|GO:0006351\|GO:0008284\|GO:0006468\|GO:0030857\|GO:0000122\| | cl.5 |
| NONMMUG025454 | chr2\|164564181\|164568494\| | NA | cl.5 |
| NONMMUG039448 | chr7\|130981670\|130982443\| | NA | cl.5 |
| NONMMUG040425 | chr8\|40917870\|40918693\| | NA | cl.5 |
| NONMMUG030591 | chr4\|125007480\|125008525\| | NA | cl.5 |
| NONMMUG016590 | chr16\|45128574\|45130278\| | GO:0008150\|GO:0007049\|GO:0016310\|GO:0006468\|GO:0043066\|GO:0007275\|GO:0030154\|GO:0008152\|GO:0016055\|GO:0016567\| | cl.5 |
| NONMMUG007704 | chr11\|102619507\|102624416\| | GO:0008150\|GO:0006508\|GO:0007165\|GO:0007049\|GO:0008152\|GO:0016310\|GO:0006887\|GO:0007155\|GO:0006810\|GO:0001701\| | cl.5 |
| NONMMUG007005 | chr11\|82963864\|82964850\| | NA | cl.5 |
| NONMMUG012805 | chr14\|46386996\|46390535\| | NA | cl.5 |
| NONMMUG007265 | chr11\|90387923\|90389590\| | NA | cl.5 |
| NONMMUG035958 | chr6\|88849814\|88874377\| | NA | cl.5 |

| | | | |
|---|---|---|---|
| NONMMUG045433 | chrX\|98148769\|98149399\| | GO:0055114\|GO:0008152\|GO:0005975\|GO:0006006\|GO:0006739\|GO:0006098\|GO:0008150\|GO:0016310\|GO:0046777\|GO:0051289\| | cl.5 |
| NONMMUG015487 | chr15\|100311187\|100361391\| | GO:0008150\|GO:0006351\|GO:0006355\|GO:0008152\|GO:0007275\|GO:0007155\|GO:0006810\|GO:0000122\|GO:0055085\|GO:0045944\| | cl.5 |
| NONMMUG045826 | chrX\|133931394\|133931811\| | NA | cl.5 |
| NONMMUG039675 | chr7\|142655442\|142661205\| | NA | cl.5 |
| NONMMUG016555 | chr16\|43627300\|43630068\| | GO:0006810\|GO:0015031\|GO:0030163\|GO:0000209\|GO:0045806\|GO:0051865\|GO:0070086\|GO:0046755\|GO:0007155\|GO:0032321\| | cl.5 |
| NONMMUG037956 | chr7\|45102835\|45103795\| | NA | cl.5 |
| NONMMUG029347 | chr4\|57073300\|57120060\| | NA | cl.5 |
| NONMMUG012067 | chr13\|117274565\|117278273\| | GO:0043066\|GO:0006954\|GO:0001568\|GO:0007275\|GO:0008284\|GO:0043410\|GO:0070371\|GO:0030154\|GO:0007155\|GO:0001525\| | cl.5 |
| NONMMUG036541 | chr6\|124530911\|124542310\| | GO:0045087\|GO:0006958\|GO:0006508\|GO:0006956\|GO:0055114\|GO:0008150\|GO:0008152\|GO:0045454\|GO:0006662\|GO:0007596\| | cl.5 |
| NONMMUG022505 | chr2\|30279631\|30281854\| | NA | cl.5 |
| NONMMUG025468 | chr2\|164846813\|164856796\| | NA | cl.5 |
| NONMMUG037985 | chr7\|45475630\|45479931\| | NA | cl.5 |
| NONMMUG004004 | chr10\|75780841\|75781981\| | NA | cl.5 |
| NONMMUG039674 | chr7\|142650770\|142653030\| | NA | cl.5 |

| | | | |
|---|---|---|---|
| NONMMUG023510 | chr2\|76595948\|76600429\| | NA | cl.5 |
| NONMMUG005875 | chr11\|44617317\|44617773\| | GO:0008150\|GO:0006355\|GO:0006351\|GO:0007165\|GO:0045944\|GO:0007155\|GO:0006810\|GO:0045087\|GO:0042113\|GO:0006954\| | cl.5 |

**NA, not applicable**

**Supplementary Figure 1. Overview of the methods and procedures to identify biomarkers**

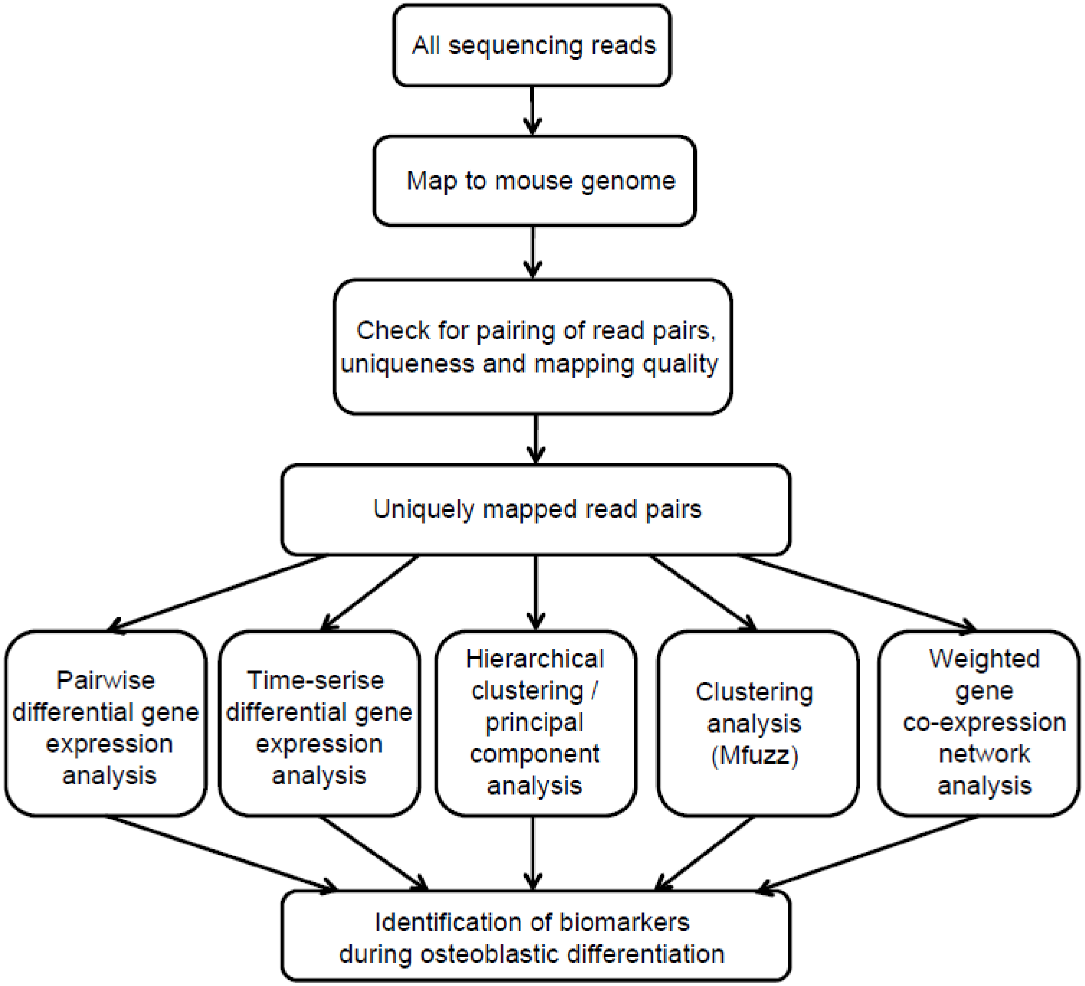

**Supplementary Figure 2. Proportion of variance for principal components**

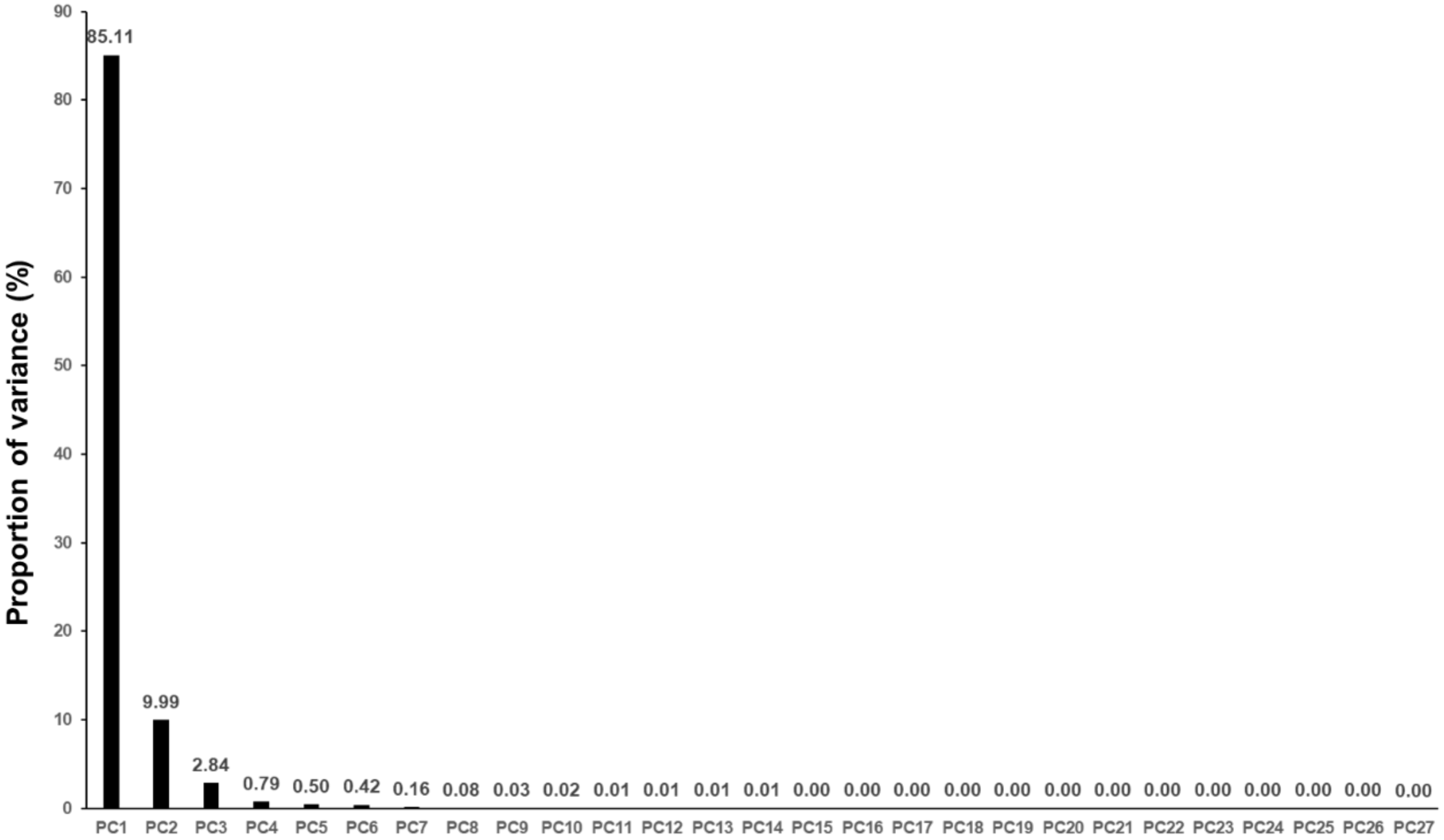

**Supplementary Figure 3. Expression profiles of known mature osteoblast markers**

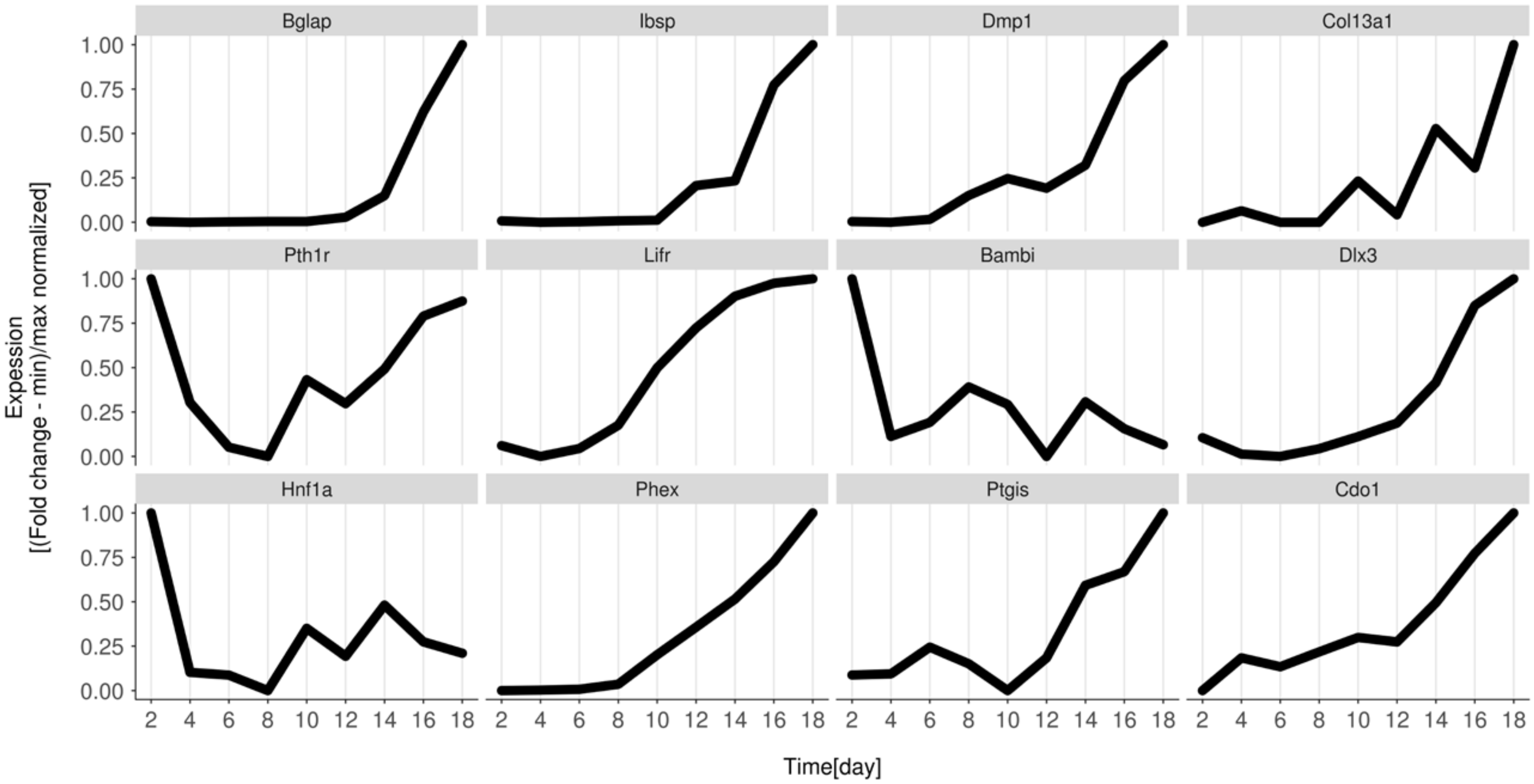

Supplement: Supplementary Materials — Supplementary Table 1: functional annotation of upregulated genes on day 18 (day 2 versus day 18). Supplementary Table 2: functional annotation of downregulated genes on day 18 (day 2 versus day 18). Supplementary Table 3: known Ensembl genes potentially associated with osteoblast differentiation. Supplementary Table 4: enriched GO terms of known Ensembl genes potentially associated with osteoblast differentiation. Supplementary Table 5: lncRNAs (NONCODE v4) potentially associated with osteoblast differentiation. Supplementary Figure 1: Overview of the methods and procedures to identify biomarkers. Supplementary Figure 2: proportion of variance for principal components. Supplementary Figure 3: expression profiles of known mature osteoblast markers. [file 7691794.f1.pdf]
